# Managing Customer Citizenship Behavior in Aviation Sector Through Relational Benefits: Mediating Role of Relationship Quality

**DOI:** 10.3389/fpsyg.2022.917434

**Published:** 2022-09-01

**Authors:** Shahzad Hassan, Norazah Mohd Suki

**Affiliations:** Othman Yeop Abdullah Graduate School of Business, Universiti Utara Malaysia, Kuala Lumpur, Malaysia

**Keywords:** customer citizenship behavior, relationship quality, altruistic benefits, socialization benefits, confidence benefits, self-expression benefits

## Abstract

The aim of this research is to investigate the mediating role of relationship quality in the relationship between relational benefits and customer citizenship behavior. Data were gathered through a systematic sampling from 334 passengers. A Survey technique was used to collect the data from respondents from multiple airports. Data were analyzed through partial least square structural equation modeling (PLS-SEM) using SmartPLS 3.3. The results of the study reveal that altruistic benefits, confidence, and self-expression benefits have a positive relationship with relationship quality while socialization benefits have a non-significant relationship with relationship quality. Similarly, relationship quality mediates the relationship between altruistic benefits, confidence and self-expression benefits, and customer citizenship behavior while relationship quality does not mediate the relationship between socialization benefits and customer citizenship behavior. This study uncovers the relational benefits and its role in the generation of customer citizenship behavior in the aviation sector and the role of relationship quality that could help managers to cultivate the benefits of customer citizenship behaviors.

## Introduction

Formerly, the aviation industry has generated $472 billion worldwide. Total revenues dropped from $472 billion to $373 during the pandemic. At the time, the situation looks favorable, and estimated revenues are predicted as $658 billion in 2022 (IATA, 2021). The pandemic situation hit the aviation industry drastically worldwide. In the year of 2020, a significant drop in travelers was seen worldwide (IATA, 2021). According to Tan (2020), COVID-19 has created the worst situation in the tourism and airline industry due to travel restrictions. In Pakistan as elsewhere, the pandemic situation significantly suppressed industry revenues but has seen a positive trend in the post-pandemic situation due to vaccination. In response to the COVID-19 situation, the airlines have updated their operational procedures and searched for alternative revenue streams (Thepchalerm and Ho, 2021) by attracting customers.

In the cut-throat competition, the birth of new airlines is increasing that are trying to grab others’ market share domestically. In this situation, the aviation sector attracts customers by engaging through customer empowerment, i.e., e-ticketing, online bookings, and self-check-in (Li et al., 2015). Customers are obliged to such airlines and intentionally suggest others whenever an individual plans to visit anywhere. Contrary, in Pakistan, passengers are shifting from local airlines to international airlines (Iqbal and Badshah, 2016). An open sky policy is deemed to allow worldwide aircraft to function from cities where domestic carriers do not work. In this situation, attracting and retaining customers increase the number of customers who foster and strengthen relationship quality. A recent study found that the aviation sector of Pakistan is suffering from customer turnover and small profits (Raza et al., 2020). Customers are switching to alternative service providers when they are dissatisfied which weakened relationship quality that can be gained through intimacy (Nobre and Simões, 2019), partner quality (Papista and Dimitriadis, 2019), passion (Ahn and Back, 2019; Nobre and Simões, 2019), self-connection (van der Westhuizen, 2018; Nobre and Simões, 2019), and, commitment (Giovanis and Ozdamar, 2016; Osobajo and Moore, 2017; Nobre and Simões, 2019) that leads customers toward citizenship behaviors (van Tonder et al., 2020). Customer citizenship behavior is a relatively new construct and a less researched area of marketing. A few studies have been conducted particularly to explore the antecedents of customer citizenship behavior with regard to customer-perceived benefits: namely functional benefits, hedonic benefits, and rational benefits. The mechanism of the relationship between customer benefits and customer citizenship behavior is not clear. Hence, this study intends to examine the mediating role of relationship quality in the relationship between customers’ perceived benefits and customer citizenship behavior. Leading toward the context-specific industry, i.e., “Pakistan aviation,” customer citizenship behavior has become critical for the survival of local airlines because of hyper-competition and improved services by other international airlines. According to a study by Zhang and Graham (2020), aviation is an essential sector of the economy which has significant financial contributions to the economy.

## Literature Review

### Customer Citizenship Behavior

That customers are “good soldiers,” the idea presented by Groth (2005), has been studied by researchers since then. Groth proposed this idea in the context of service firms, the theme of that was that customers may perform voluntary behaviors for the organization which are not essential for the success of the exchange process (between customer and service provider), but these are extra roles played by the customer which help the service providing organization. Studies on customer citizenship behavior have been published in high-impact journals with a comparatively higher number of readers and citations. But there exists confusion and misunderstanding on the construct. According to recent research by Gong and Yi (2019), customer citizenship behavior is misunderstood by many researchers because many of them failed to distinguish this construct from similar concepts like value co-creation, customer participation, and customer engagement. According to some recent studies, because of changing behavioral situations and trends, the phenomenon should be reconsidered covering such diverse behaviors (Ajiboye et al., 2019; Davenport, 2020; Rust, 2020). Many studies have been done recently which integrate the customer citizenship behavior with the concepts like value co-creation (Gong and Yi, 2019) and behavioral loyalty (Kim M. G. et al., 2014; Kim M. J. et al., 2014; Lee et al., 2014; Han et al., 2019).

### Relationship Quality

In this hyper-competitive market, an organization’s long-term and profitable relationship with customers is essential. In relationship marketing, this construct is well defined. According to Smith and Krannich (1998), “relationship quality is variety of positive relationship and reflection of the overall evaluation of relationship.” According to Hennig-Thurau and Klee (1997) “relationship quality is the degree of appropriateness of a relationship to fulfill the needs of customer associated with that relationship.” High relationship quality indicates that the customer has high trust in the product or service of the firm and that higher trust is the outcome of satisfactory past performance (Kim and Han, 2008). Relationship quality is having dimensions like trust and satisfaction (Kim and Han, 2008). Customer satisfaction has been studied, defined, and conceptualized by many researchers as an evaluation process (Ryu et al., 2008). Trust is defined as “Willingness to rely on an exchange partner in whom one has confidence” (Moorman, 1993; Moorman et al., 1993a,b). Likewise, Fournier (1998) has identified five dimensions of relationship quality i.e., commitment, self-connection, love and passion, intimacy, and partner quality. According to Nobre and Simões (2019), Commitment is the psychological attachment of individuals inclined to have long-term relationships. According to Ahn and Back (2019), partner quality is the measure of the increasing strength of the relationship between customer and the organization. Bonding which is made by consumers is termed love and/or passion (Nobre and Simões, 2019). This bonding is basically a feeling of a consumer toward the organization. Intimacy is the eagerness of a customer to share information with the firm which is providing a product or service (Nobre and Simões, 2019). Lastly, self-connection is defined as “the profound and solid ties that evoke identify system” [*sic*] (Nobre and Simões, 2019). The present research study uses relationship quality as a three-dimensional construct i.e., commitment, partner’s quality, and love/passion for a thorough analysis of the relationship of these dimensions with customer citizenship behavior. Relationship quality is a well-studied construct and it is widely studied with consumer behavior.

### Relationship Quality and Customer Citizenship Behavior

No doubt customer citizenship behavior is theoretically linked with these concepts, and CCB is connected with the domain of relationship marketing. The link between customer citizenship behavior and relationship marketing was proposed by Gruen (1995), which basically focuses on customer citizenship behavior as an outcome of an organization’s relationship with the customer. So, from this discussion it can be argued that customer citizenship behavior can be seen in the context of the customer-organization relationship as Bowen (1990) described it as a form of social exchange. According to Barry and Terry (2008), relationship quality is a critical determining factor for a long-term relationship between an organization and its customer. Groth (2005) alleged that customer trust and customer satisfaction (dimensions of relationship quality) lead the customers to show behaviors like recommendations, providing positive feedback, and helping other customers (a dimension of customer citizenship behavior). It was found by many studies that the exhibition of customer citizenship behavior is strongly influenced by the quality of the relationship between customer and service provider. According to, customers having a good quality relationship with their service provider are more likely to feel good and engage in citizenship behavior. As citizenship is basically consumer behavior and customer citizenship behavior has dimensions like recommendations, feedback, and helping behavior (Chang and Jung, 2017). Kim and Han (2008) alleged that the relationship quality is an important predictor of loyalty intention and these loyalty intentions include the intention to recommend, which is the dimension of customer citizenship behavior. In another study of, it is alleged that relationship quality plays a critical and important role in forming the recommendation of product or service in the hotel industry. So, it can be hypothesized that customer citizenship behavior depends largely upon the relationship quality.

**H1:** Relationship quality has a positive relationship with customer citizenship behavior in the aviation industry.

### Relational Benefits

Rational benefits are initially conceptualized by Gwinner et al. (1998) as a multidimensional construct. Before this, relational benefits were considered unidimensional in nature, as Morgan and Hunt (1994) originally conceptualized this construct. The main theme of providing relational benefits to the customers was to give the loyal customers three kinds of benefits, i.e., confidence, social, and special treatment. For example, reducing the ambiguities about the organization enhances the confidence, making friendship is a social joy, and suggesting better deals that cost less to customers is special treatment. Relational benefits are basically part of overall customer benefits, although this concept is being studied and researched for more than two decades (Hult and Ferrell, 2012), there still exists misunderstanding. Overviews and researches in different studies about the relational benefits provide different outcomes (Hennig-Thurau et al., 2002; Meldrum and Kaczynski, 2007; Verma et al., 2016).

### Relational Benefits and Relationship Quality

According to a study by Verma et al. (2016), relationship quality mediates the relationship between relational benefits and customer loyalty. Rational benefits and relationship quality are two important approaches in which the prior approach promises the benefits of a relationship in the future, and the second approach emphasizes the “degree of appropriateness” of relationships (Hennig-Thurau and Klee, 1997). Prior studies have tested the impact of relational benefits on relationship quality with the themes including confidence, economic, social, and exclusive treatment benefits as suggested by Gwinner et al. (1998), but past studies have focused less on the themes as the best of knowledge that included self-expression, and altruistic benefits as dimensions of relational benefits motivated by Papista and Dimitriadis (2019). Number of studies found the positive relationship between relational benefits and relationship quality (Fitria et al., 2016; Blazquez-Resino and Gołąb-Andrzejak, 2017; Akrout and Nagy, 2018). The same relationship has been tested in the natural cosmetics industry (Papista and Dimitriadis, 2019).

The aim of the present research study is to integrate these two-research streams (i.e., Relational benefits and relationship quality) and develop a comprehensive model. Specifically, the present study proposed the mediating role of relationship quality between the relationship of relational benefits and customer citizenship behavior. For this purpose, the philosophy of relationship marketing is essential to explain comprehensively. According to Zeithaml et al. (2018), relationship marketing is the “philosophy of conducting a strategically oriented business with a focus of maintaining and improving the quality of relationships with current customers, rather than focusing on the efforts of acquiring new customers.” The relationship marketing strategy focuses on one service provider to another (Gummerus et al., 2017). As it is stated earlier that relationship marketing, is based on two approaches namely the relational benefits and relationship quality; the relational benefits approach is based on the assumption that customers can be benefited by long-term relationships with a service provider and the service provider can also benefit (Chien-Jung, 2017; Fatima and Mascio, 2020). The following hypotheses have been generated on the basis of the literature review:

**H2:** Confidence benefits have a positive relationship with relationship quality in the aviation industry.

**H3:** Self-expression benefits have a positive relationship with relationship quality in the aviation industry.

**H4:** Social benefits have a positive relationship with relationship quality in the aviation industry.

**H5:** Altruistic benefits have a positive relationship with relationship quality in the aviation industry.

### Relationship Quality as Mediator

Social exchange theory is a fundamental theory that provides a basis for an explanation of the exchange association between two parties. According to Blau (1968), social exchange theory assumes that two parties have a relationship with each other with the presumption that by keeping this relationship, they both will be remunerated in the future (Hunt and Morgan, 1994a,b; Hunt and Morgan, 1995). Fournier (1998), also explained social exchange theory as “association between dyad partners involved a reciprocal exchange.” The service organizations tend to provide more benefits to their customer with whom they wish for good relationships and customers tend to keep stronger connections with those organizations and have intentions to assist through positive feedback and recommendation. In the case of relationship quality, customers show a high level of satisfaction, psychological commitment, trust, and attachment with the organization, and all these motivations create positive behaviors i.e., helping the organization and other customers (Kim et al., 2008; Xie et al., 2017). Prior studies identified customer behaviors are influenced by the relationship quality (Su et al., 2016). Relationship quality influences the intentions of customers to share information with the organization (Wu and Cheng, 2018), support for product or service (Tajvidi et al., 2021), and give a positive word of mouth (Jin et al., 2013). These roles are not essential for the delivery of service, rather these are extra roles performed by the customer for the organization, and these extra- roles are related to customer citizenship behavior (Nguyen et al., 2014). Another, relatively latest research study conducted by van Tonder et al. (2020), concluded that commitment, satisfaction and trust dimensions of relationship quality are critical to producing customer citizenship behavior. Similarly, the dimensions of customer citizenship behavior e.g., helping other customers, positive feedback, and recommendations are dependent upon relationship quality (Kim and Han, 2008).

In the literature on service marketing, researchers have not paid attention to the association between relational benefits and customer citizenship behavior mediated by relationship quality. Prior research has shown the effects of relational benefits on relational results such as cross buying and word of mouth (Nyffenegger et al., 2015; de Oliveira Santini et al., 2018) but the effect of relational benefits on customer citizenship behavior when mediated by relationship quality, is ignored. The studies have shown positive results of measurements of relational benefits such as confidence, social, self-expressive, and altruistic on relationship quality (Chou and Chen, 2018; Papista and Dimitriadis, 2019) which leads to an increase the customer citizenship behavior. Moreover, it is found that the relationship between relational remunerations and customer citizenship behavior with a mediating effect on relationship quality has been found in the natural cosmetic industry (Papista and Dimitriadis, 2019). According to another research study, customers that develop confidence in a service-providing organization based on past experiences may have a strong cognitive reason to strengthen relationship quality (Berry, 1995). In a similar way, customers are attracted to services or products that symbolically express their personalities. Those customers are conscious buyers who are deemed to fulfill their standards or to generate symbolic recognition (Butt et al., 2017). Service organizations enhance the relationship by providing services or products that must match consumers’ belief systems which ultimately fosters buying behavior in a form of recommendations (Stathopoulou and Balabanis, 2016). Concerning social benefits, the social bonds between clients and the service providers lead to a higher level of commitment which made the relationship quality strong, and customers are engaged in a citizenship behavior (Wei et al., 2015). On the basis of this discussion, the following hypotheses have been made:

**H6:** Relationship quality mediates the relationship between confidence benefits and customer citizenship behavior in the aviation industry.

**H7:** Relationship quality mediates the relationship between self-expression benefits and customer citizenship behavior in the aviation industry.

**H8:** Relationship quality mediates the relationship between social benefits and customer citizenship behavior in the aviation industry.

**H9:** Relationship quality mediates the relationship between altruistic benefits and customer citizenship behavior in the aviation industry.

## Theoretical Framework

**FIGURE 1:**
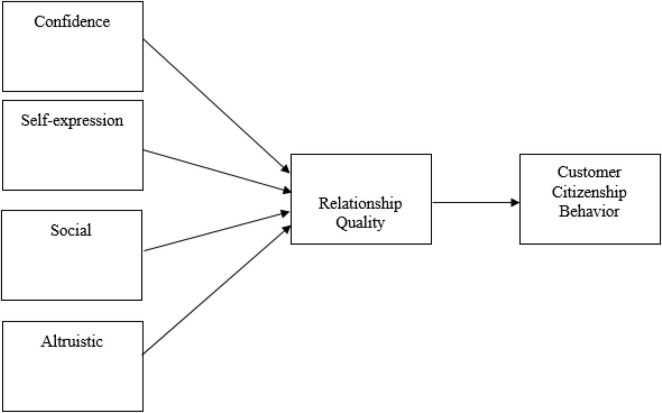
Theoretical framework.

This research includes four independent variables (see Figure 1) that are the part of relationship benefits. In the research framework, Confidence, Self-expression, Social benefits and Altruistic benefits are independent variables while relationship quality is the mediator and customer citizenship behavior is the dependent variable of the study.

### Methodology

The present research study is inclined to examine the relationship between customer-perceived relational benefits namely (confidence, social, self-expression, and altruism) and customer citizenship behavior with a mediating role of relationship quality. This is a cross-sectional study—the data was collected and analyzed at one point in time throughout the sample population. Due to time and resource constraints, we conducted a cross-sectional study because it is less time-consuming as compared to a longitudinal study.

### Instrumentation

Customer citizenship behavior is a dependent variable of the study that is operationally defined as “customer recommendation of the service to others, providing feedback to the service organization for improvements, and, assisting other clients with regard to problems encountered in service delivery” (Groth, 2005). A 12 item multi-dimensional scale was adapted to measure customer citizenship behavior, which includes recommendations, feedback, and helping customers (Groth, 2005). Same as relationship quality was considered as a mediator variable of the study. Fournier (1998) operationally defined relationship quality as a multifaceted construct comprised of service-related components such as affective and socio-motive connections (love/passion and self-connection), behavioral ties (commitment), and strong cognitive convictions (intimacy and partner quality). A total number of 17 items was adapted to measure the five dimensions of relationship quality from the studies of Papista and Dimitriadis (2019).

Relational benefits are operationalized through dimensions such as confidence benefits, self-expression benefits, socialization benefits, and altruistic benefits. The 17 item scale is adapted to measure relational benefits (confidence benefits, self-expression benefits, socialization benefits, and altruistic benefits) from the study of Papista and Dimitriadis (2019). All items were measured on a seven-point Likert type scale (1 = Strongly Disagree to 7 = Strongly Agree).

### Population and Sampling

The target population of this study is the customers of the aviation industry of Pakistan that are using the services of public and private-owned local airlines operating within the country. A total number of 27 airlines operate in Pakistan, which include 4 local airlines. The remaining 23 airlines are international (CAAPakistan, 2019). All 4 local airlines entertained 7.42 million passengers in 2019, with which terminal passengers were 6.005 (99.75%) and transit passengers were 0.015 (0.246%). The details of passengers and services of these airlines have been obtained from the annual report issued by the Pakistan Civil Aviation Authority (PCAA) in the financial year 2019 (CAAPakistan, 2019). Based on the given population, the sample size was determined using the formula given by Mendenhall et al. (1993). Hence, 334 samples were considered sufficient to be representative. To tackle the issue of sample attrition, the sample size was increased to 477 based on past studies.

A Systematic (probability) sampling design has been used to take data from respondents from four airports of Lahore, Faisalabad, Multan, and Sialkot.

Data has been analyzed by both descriptive and inferential statistics. Partial least square structural equation modeling (PLS-SEM) has been used to access the relationships among the variables.

## Demographic Profile

Respondents were selected from the aviation sector of Pakistan to indicate a number of various aspects that are related to demography which included gender, age, income, occupation, qualification, airline type, airline name, city, and destination.

The sample was taken from the airports of Pakistan located in the four provincial capitals namely, Lahore, Faisalabad, Sialkot, and Multan (see Table 1). Demographic details are mentioned in Table 2 Samples collected from the aviation sector consisted of 284 (82.3) male respondents, and 50 (17.60%) female respondents. As far as the ages of passengers are concerned, 131 (39.22%) respondents fall in the age bracket of 16–30, 144 (43.11%) respondents fall in the age bracket of 31–45, 50 (14.97%) fall in 46–60, and 9 (2.6%) respondents fall in the age category of 61–75, respectively. In this regard 136 (40.7%) respondents from the data set have a bachelor’s degree, 169 (50.59%) have master’s, and only 29 (8.68%) respondents had neither. Also, the occupation of respondents was asked to assess the notion that whether there is a large number of businesspeople who travel through airlines or job holders have also the tendency to travel through an airline. In this way, 115 (34.43%) respondents were reported as business people while 219 (65.56%) respondents were those who work somewhere. Analysis revealed that 121 (36.22%) of the respondents had an income level of Rs. 30,000–60,000, while 126 (37.72%) of the respondents belonged to the category having an income level of Rs. 61,000–80,000. Furthermore, 87 (26.04%) of the respondents were those who belonged to the other category.

**TABLE 1:** Summary of airports and passengers.

Airports	Passengers	Percentage	Distribution	Questionnaires
Lahore	996,266	79.62%	477 × 79.62/100 = 379.7	380
Faisalabad	102,049	8.15%	477 × 8.15/100 = 38.8	39
Sialkot	32,304	2.5%	477 × 2.5/100 = 11.9	12
Multan	120,647	9.64%	477 × 9.64/100 = 45.98	46
Total	1,251,266	100%	477.49	477

**TABLE 2:** Demographic profile.

Item	Frequency	Percentage
**Gender**		
Male	284	82.30%
Female	50	17.60%
**Age**		
16–30	131	39.22%
31–45	144	43.11%
46–60	50	14.97%
61–75	9	2.60%
**Qualification**		
Bachelor	136	40.70%
Master	169	50.59%
Other	29	8.68%
**Occupation**		
Business	115	34.43%
Work	219	65.56%
**Income**		
Rs. 30,000–	121	36.22%
Rs. 60,000		
Rs. 61,000–	126	37.72%
Rs. 80,000		
Other	87	26.04%
**Airline name**		
PIA	123	36.82%
Serene air	11	3.29%
Airblue	39	11.67%
Other	161	48.20%
**Destination**		
Outside of country	266	79.30%
Inside of country	68	20.35%
**City**		
Lahore	135	40.41%
Faisalabad	129	38.62%
Sialkot	33	9.88%
Multan	37	11.07%

Similarly, respondents were requested to select an airline from the category for which they used the services when making a trip. In total 123 (36.82%) of the respondents selected PIA, 11 (3.29%) respondents selected Serene Air, 39 (11.67%) of the respondents Air blue, and 161 (48.20%) of the respondents used other airlines for their travel. With regard to the destinations, it is revealed from the information collected from individual passengers that 266 (79.3%) of the respondents have made the country tour while 68 (20.30%) of the respondents have made the country tour. The respondents in the current study were requested to select from the category of cities which revealed that 135 (40.41%) of the respondents belonged to Lahore, 129 (38.62%) of the respondents belonged to Karachi, 33 (9.88%) were from Peshawar, and 37 (11.07%) of the respondents were from Quetta.

The first step in the PLS-SEM analysis is the evaluation of the outer or measurement model. This model deals with component measurement, which actually determines or defines how well indicators or items load tentatively and are linked with the respective constructs. The analysis of the measurement models checks that survey items accurately measure the variables they were designed or made to measure, thus confirming that they are not only reliable but also valid (Hulland, 1999; Ramayah et al., 2011; Hair et al., 2013). To begin, internal consistency is a term used to describe the consistency of a result across multiple test items. Consequently, in this study, internal consistency and reliability were measured by investigating CR. According to Hair et al. (2013), contrasting Cronbach’s alpha, CR does not assume the equal-indicator construct loading. CR varies between 0 and 1; the threshold value would not be less than 0.6 (Henseler et al., 2009), but a value of 0.7 or above is the most desirable (Hair et al., 2012). Hence, CR values between 0.6 and 0.7 show average internal consistency, while values between 0.7 and 0.9 are more adequate (Nunnally and Bernstein, 1994). Consequently, in this research, values of CR and Cronbach’s alpha for all variables were analyzed, and the results are shown in Table 3, which indicates that all values of CR and Cronbach’s alpha exceeded the suggested threshold value of 0.70 (Henseler et al., 2009).

**TABLE 3:** Labeling, loadings, composite reliability (CR), average variance extracted (AVE), and Cronbach’s alpha.

First order	Second order	Items	Loading range	AVE	CR	Alpha
Recommendation		CCB1–CCB4	0.854–0.921	0.807	0.943	0.920
Feedback		CCB5–CCB8	0.859–0.911	0.781	0.934	0.906
Helping behavior		CCB9–CCB12	0.829–0.896	0.739	0.919	0.882
		Recommendation	0.862			
		Feedback	0.908			
		Helping behavior	0.853			
	Customer citizenship behavior			0.765	0.904	0.937
Partner quality		RQ1– RQ2	0.971–0.973	0.945	0.972	0.942
Commitment		RQ3–RQ7	0.867	0.780	0.947	0.930
Passion/love		RQ8–RQ10	0.880–0.927	0.815	0.929	0.886
Intimacy		RQ11–RQ12	0.933–0.940	0.877	0.934	0.859
Self-connection		RQ13–RQ17	0.812–0.908			
		Partner quality	0.741			
		Commitment	0.901			
		Passion/Love	0.899			
		Intimacy	0.833			
		Self-connection	0.839			
	Relationship quality			0.713	0.925	0.957
Confidence benefits		RB1–RB5	0.812–0.896	0.775	0.945	0.927
Socialization benefits		RB6–RB9	0.848–0.919	0.806	0.943	0.920
Self-expression benefits		RB10–RB14	0.804–0.932	0.787	0.949	0.932
Altruistic benefits		RB15–RB17	0.8530.916	0.799	0.922	0.873
		Confidence benefits	0.852			
		Socialization benefits	0.913			
		Self-expression benefits	0.904			
		Altruistic benefits	0.712			
	Relational benefits			0.721	0.911	0.953

The values of CR in this research ranged from 0.904 to 0.972, showing the reliability of the measurement model. The next one is convergent validity, which is defined as the degree to which measures of the same variables are related to each other on a theoretical basis (Henseler et al., 2009). Therefore, it indicates the extent of correlation between measures of the same construct (Hair et al., 2013). To recognize the convergence element in the measurement of a construct, AVE is employed with a threshold value of 0.50 and above (Henseler et al., 2009; Hair et al., 2012). The AVE value (0.50) shows average or acceptable convergent validity. In this research, convergent validity was evaluated by examining the values of AVE. Results in Table 3 indicate that the AVE values of all variables exceed the threshold value of 0.5 (Henseler et al., 2009; Hair et al., 2012). The results indicate that values of AVE range from 0.807 to 0.945, hence it can be concluded that the convergent reliability is proven.

One of the most conventional methods for evaluating discriminant validity is the Fornell-Larcker criterion (Hair et al., 2013). Discriminant validity is recognized when the square root value of the AVE of each variable is greater than the highest correlation of that variable with any other variable (Henseler et al., 2009; Hair et al., 2013). Hence, in this research, discriminant validity was evaluated by comparing the square root of AVE for every variable with the correlations presented in the correlation matrix. Table 4 indicates the results of the Fornell-Larcker Criterion assessment with the square of variables. The square root of AVE is higher than its maximum correlation of constructs with the other constructs. As a result, it is concluded that the variable’s discriminant validity has been demonstrated (Henseler et al., 2009; Hair et al., 2013).

**TABLE 4:** Discriminant validity and correlation.

	1	2	3	4	5	6
1. Altruistic benefits	0.894					
2. Confidence benefits	0.442	0.861				
3. Customer citizenship behavior	0.466	0.650	0.870			
4. Relationship quality	0.735	0.496	0.554	0.844		
5. Self-expression benefits	0.638	0.620	0.595	0.707	0.887	
6. Socialization benefits	0.512	0.779	0.636	0.571	0.763	0.898

In this research, systematic-model analysis of structural models was carried out to give a clear view of the results and to test the hypothesis. The assessment of the inner model starts with the evaluation of direct relationships among independent and dependent variables. The size of the path coefficient was evaluated by the PLS-SEM algorithm, and the significance of the relationship was tested by the PLS-SEM bootstrapping method in SmartPLS 3.0. Henseler et al. (2009) and Hair et al. (2011, 2012, 2013). The first model focused on direct relationship analysis among independent and dependent variables (H1–H4). Then, we focused on the mediator variable, the analysis of the relationship between independent and mediating variables (H5). Then, the relationship between the mediating and dependent variables was examined. Moreover, in the model, the mediation-analysis tool was placed where H6–H9 were examined. According to the results shown in Table 5, AB has a positive impact on RQ (0.0.483; *t* = 10.226); so, H1 is supported. Likewise, results show the positive impact of CB on RQ (0.0.021; *t* = 3.836); therefore, H2 is supported. The results indicate a significant and positive effect of SEB on RQ (0.063; *t* = 5.252), so H3 is supported. H4 is not supported because results indicated no significant effect of SB on RQ (0.0.021; *t* = 0.162). According to the results, RQ has a significant positive impact on CCB (0.057; *t* = 12.671), therefore H5 is supported.

**TABLE 5:** Regression analysis.

Direct and indirect paths	Beta	S.E	*t*-test	LLCI	ULCI	Decision	VIF	*f* ^2^	*R* ^2^
AB → RQ	0.483	0.046	10.226	0.410	0.511	Supported	1.695	0.368	0.487
CB → RQ	0.021	0.014	3.836	0.006	0.006	Supported	2.563	0.003	
SEB → RQ	0.363	0.069	5.252	0.270	0.432	Supported	2.988	0.123	
SB → RQ	0.021	0.046	0.162	–0.024	0.036	Not supported	3.765	0.000	
RQ → CCB	0.557	0.044	12.671	0.487	0.582	Supported	1.000	0.444	
CB → RQ → CCB	0.030	0.009	3.398	0.003	0.003	Supported			
SB → RQ → CCB	0.004	0.025	0.165	–0.015	0.018	Not supported			
SEB → RQ → CCB	0.202	0.045	4.481	0.144	0.245	Supported			
AB RQ CCB	0.263	0.034	7.844	0.239	0.268	Supported			

*AB, Altruistic Benefits; CB, Confidence Benefits; SEB, Self-expression Benefits; SB, Socialization Benefits; RQ, Relationship Quality; CCB, Customer Citizenship Behavior.*

According to the results, the path coefficient among three independent variables and the mediating variables is positive, but one independent variable does not have a positive path coefficient. H6 is supported by the findings, and RQ mediates the relationship between CB and CCB (0.030; *t* = 3.398). RQ does not mediate the relationship between SB and CCB (0.004; *t* = 0.165), so H7 is not supported. Regarding H8, the findings show that RQ mediates the relationship between SEB and CCB (0.202; *t* = 4.481), indicating that H8 is supported. Similarly, results indicate that RQ mediates the effect of AB on CCB (0.263; *t* = 7.844), therefore H9 is also supported.

## Discussion

Relational benefits and relationship quality play a key role in promoting favorable consumer behavior. In this study, a comprehensive model had been proposed to understand in a better way the relationship success between service providers and customers.

The first hypothesis of the present study was that “relationship quality has a positive relation with customer citizenship behavior in an aviation industry.” Consistent with the previous studies (Xie et al., 2017; Choi and Lotz, 2018, p. 614; van Tonder et al., 2020), the results of the current study supported the notion that relationship quality positively affects customer citizenship behavior. When the service provider develops a strong relationship, customers tend to perform citizenship behavior in a form of recommending the service provider, giving feedback to the service provider, and helping other customers during service encounters or service usage. Hence, the first hypothesis of the study was accepted.

“Relational benefits have a positive influence on relationship quality in an aviation industry.” The results of the study have supported this statement and are reliable with the past research as well (Papista and Dimitriadis, 2019). The relational benefits resulted from a long-standing relationship with the service provider and customers are willing to get these benefits in a form of altruistic, self-expression, confidence, and socialization benefits in the firm’s offerings. Hypothesis 2 was “Confidence benefits have a positive relationship with relationship quality in the aviation industry,” hypothesis 3 was “Self-expression benefits have a positive relationship with relationship quality in the aviation industry,” hypothesis 4 was “Social benefits have a positive relationship with relationship quality in the aviation industry,” and hypothesis 5 was “Altruistic benefits have a positive relationship with relationship quality in the aviation industry.” From the results of the study, these hypotheses have been accepted, except hypothesis 4, which was not supported. There can be possible reasons for this rejection. The previous studies where this relationship was supported were conducted in different industries, and different geographical locations for example studies have been conducted in the mobile phone industry (Hsu and Liou, 2017), and the social media industry, where the target population is more interested in obtaining entertainment, pleasure, emotional attachment, and fun from a service or a product rather than anything else. Also, the cause of the rejection of this relationship in the present study may be the context-specific (Pakistan aviation industry) airline industry where people are more concerned about the monetary benefits rather than socialization.

The analysis of the four direct relationships in the study indicates that relationship quality is important to consider for the service providers if they wish to acquire more customers because if the customers are satisfied and remained committed to the service organization, they also reciprocate in a form of performing client citizenship behavior for the service provider. “Relationship quality mediates the relationship between relational benefits and customer citizenship behavior in an aviation industry.” Consistent with the prior studies in the literature (Wei et al., 2015; Verma et al., 2016), the current study has supported the notion that relationship quality intervenes in the relationship between relational benefits and client citizenship behavior. H6 was “Relationship quality mediates the relationship between confidence benefits and customer citizenship behavior in the aviation industry” and H7 was “Relationship quality mediates the relationship between self-expression benefits and customer citizenship behavior in the aviation industry.” Whereas H8 “Relationship quality mediates the relationship between social benefits and customer citizenship behavior in the aviation industry.” H9 was “Relationship quality mediates the relationship between altruistic benefits and customer citizenship behavior in the aviation industry.” All these indirect relationships are supported by the results of the present study except H8, which is not supported. Again the reasons may similar to as discussed earlier. As the results showed the influence of the exposure of relational benefits, it complements relationship quality and fosters customer citizenship behavior. The results largely support the proposed relationships among the variables. Data analysis supported the mediating role of relationship quality as well. The findings of the present study suggest that the constructs of confidence, self-expression benefits, and altruistism benefits as dimensions of relational benefits influence the customer citizenship behavior indirectly. In addition, the study highlights the mediating relevance of relationship quality in influencing customer citizenship behavior.

## Limitations and the Future Research

Besides the several significant contributions highlighted in the present study regarding relational benefits, relationship quality, and customer citizenship behavior in the aviation sector, it has several limitations that need to be demonstrated. This study only has focused on the customer sides of behavior, and the effort in this study was made to create a linkage of the customer benefits toward the customer behavior through relationship quality in the aviation sector. Only a handful of studies linked employees’ side behavior with the customer side. Hence, an investigation concerning the relationship between different types of employee behaviors and customer citizenship behavior through the relationship quality is needed. There are other concepts that are of a non-transactional nature and are recommended by the previous studies as well, like customer courtesy (Bove et al., 2009) or customer ethical behavior (Dang et al., 2020), displaying affiliation (Bove et al., 2009), that also affect customer behaviors such as citizenship behavior and also come within the domain of customer citizenship behavior, have not been given consideration in the present study.

This study has taken the relationship quality as a mediator of the relationship between CCB and its relational benefits. Future studies should include relationship quantity (frequency and duration) to examine the effects of such variables on CCB. The present study focused on a single service sector, the aviation sector of a single country, Pakistan. Future research should investigate the relationships across different industries and different countries to provide generalizable results. This study has also the limitation of the cross-sectional design. Future research studies can examine how the associations between customers’ perceived benefits, relationship quality, and citizenship behaviors may vary across the different stages of the relationship (Reinartz et al., 2004). So, this is another aspect that should be investigated in future studies.

## Data Availability Statement

The raw data supporting the conclusions of this article will be made available by the authors, without undue reservation.

## Ethics Statement

Ethical review and approval were not required for the study on human participants in accordance with the local legislation and institutional requirements. Written informed consent for participation was not required for this study in accordance with the national legislation and the institutional requirements.

## Author Contributions

SH contributed theoretical construction, data collection, and analysis of the data. NS performed translation and article reviews. Both authors contributed to this article and approved the version submitted.

## Conflict of Interest

The authors declare that the research was conducted in the absence of any commercial or financial relationships that could be construed as a potential conflict of interest.

## Publisher’s Note

All claims expressed in this article are solely those of the authors and do not necessarily represent those of their affiliated organizations, or those of the publisher, the editors and the reviewers. Any product that may be evaluated in this article, or claim that may be made by its manufacturer, is not guaranteed or endorsed by the publisher.
